# Burden of vaccine-preventable diseases in adults (50+) in the United States: a retrospective claims analysis

**DOI:** 10.1186/s12889-024-20145-0

**Published:** 2024-10-25

**Authors:** Maximilian Hartmann, Nathalie Servotte, Emmanuel Aris, T. Mark Doherty, Ahmed Salem, Ekkehard Beck

**Affiliations:** 1 Institute for Medical Information Processing, Biometry and Epidemiology- IBE, LMU Munich, Munich, Germany; 2 Pettenkofer School of Public Health, Munich, Germany; 3 GSK, Wavre, Belgium

**Keywords:** Adult, Burden, Clinical, Downstream, United States, Vaccine-preventable disease

## Abstract

**Background:**

In adults aged 50 + years, vaccine-preventable diseases (VPDs) pose a significant health burden and can lead to additional ‘downstream effects’ of infection beyond the acute phase e.g., increasing the risk for non-communicable disease and exacerbating chronic conditions. The aim was to understand and quantify the burden of VPD downstream effects in hospitalised adults in the United States.

**Methods:**

This retrospective observational study analysed hospitalisation claims data (2016–2019) with 1-year follow-up, in adults with a VPD diagnosis versus matched controls (using Optum’s de-identified Clinformatics Data Mart Database). Outcomes included mortality; increase in Charlson Comorbidity Index (CCI) score; new diagnosis of comorbidities; and loss of independence (defined by need for home health/home care and/or move to long-term facility).

**Results:**

Mortality was significantly increased in VPD cases versus controls at 30-day (risk ratio [RR] of 4.08 [95% CI 3.98–4.18]) and 1-year follow-up (RR 2.76 [2.73–2.80]). Over a 1-year follow-up period, morbidity increased following VPD hospitalisation: 65–86% of VPD cases had new comorbidities diagnosed (versus 13–41% of controls); with a significantly higher mean increase in CCI score versus baseline (3.23 in VPD cases versus 0.89 in controls, *p* < 0.001). Adults were observed to experience a worsening of their health status and were less likely to return to their original health state. In addition, 41% of VPD cases had a loss of independence following hospitalisation versus 12% of controls; as seen by an increased need for home assistance (in 25% versus 9% of controls) and/or a move to a long-term care facility (in 29% versus 6% of controls).

**Conclusions:**

This analysis suggests that VPD hospitalised cases suffer significantly worse clinical outcomes than controls, with downstream effects that include increased mortality and morbidity, and greater loss of independence. Evidence on potential downstream effects of infection is relatively new, and this additional burden is generally not considered in vaccine decision-making. More research is needed to disentangle the effect of VPDs on new comorbidities versus the natural course of the condition. Increasing awareness among adults, healthcare providers and decision makers could help to increase adult vaccination coverage, and reduce the clinical burden of VPDs.

**Supplementary Information:**

The online version contains supplementary material available at 10.1186/s12889-024-20145-0.

## Background

In adults aged 50 years and older, vaccine-preventable diseases (VPDs), such as influenza, pneumococcal disease, herpes zoster and pertussis, pose a significant health burden [1]. In the United States (US) alone, they are estimated to cost society around $26.5 billion annually, with substantial direct medical costs [2]. The Centers for Disease Control and Prevention (CDC) report that 70 to 85% of deaths and 50 to 70% of hospitalisations related to seasonal influenza occur in people aged 65 years and older in the US [3].

In addition to the direct illnesses and healthcare usage attributed to the acute phase of the disease, VPDs can lead to additional (and often unrecognised) disease burden from so-called ‘downstream effects’ of infection, particularly in older adults. VPDs can increase the risk for non-communicable disease and exacerbate existing chronic conditions e.g., influenza can increase the risk of myocardial infarction and stroke, and lead to exacerbations of chronic obstructive pulmonary disease (COPD) [4]. A recent descriptive analysis of US claims by Doherty et al. (2022) [5] explored potential downstream effects of VPDs in adults aged 50 years and older, by assessing outcomes in hospitalised patients admitted for a primary non-VPD but with a concurrent secondary VPD diagnosis. The study found that these patients had a longer length of hospital stay and poorer health outcomes than those without any VPD (primary or secondary diagnosis) [5]. Due to complex interactions of VPDs with frailty, morbidity and disability, patients may not recover to the same health state as before the onset of the VPD [6]. The VPD may result in permanent loss of functional ability, falls caused by weakness following a VPD, a loss of independence, cognitive decline, or even premature death [1].

The downstream effects of VPDs can have a significant impact on health and costs, and preventive vaccination has the potential to reduce this burden – for example by reducing risk of cardiovascular disease [7]. However, downstream effects are rarely studied or defined, and though evidence of association between VPD and downstream effects is very strong, evidence of the direct impact on healthcare utilisation remains scarce. Therefore, estimates of VPD burden in older adults and valuations of adult immunisation programs typically do not consider downstream effects [1]. This potentially contributes to underestimation of the true cost and burden of VPDs, and consequently, a lower perceived need to vaccinate. Improved understanding of the full burden of VPDs could inform discussions with (older) adults about the potential risks of VPDs and benefits of vaccination.

The aim of this retrospective claims database analysis was to gain further insights and to quantify the burden of VPD downstream effects in hospitalised adults aged 50 years and older in the US. Hospitalisations account for the majority of healthcare costs and are, therefore, of particular interest to healthcare payers and policy decision makers [8]. A cut off age of 50 years was chosen to match the age at which some routine adult vaccinations begin to be recommended (e.g., herpes zoster vaccination [9]) and the age considered for potential routine pneumococcal vaccination in the US [10]. This study extends the analysis of Doherty by including a 12 month follow-up period and capturing both current VPDs (diseases for which there is a licensed vaccine) and near-term VPDs (diseases for which vaccines are in phase III trials). Most importantly, the study includes a comparative analysis versus matched controls, to determine the health impact of VPDs. The objective of this paper was to assess the clinical impact (i.e., mortality, increase in Charlson Comorbidity Index [CCI] score, diagnosis of new comorbidities, and loss of independence/ change in residence status) in hospitalised patients with any VPD diagnosis (primary, secondary or both) versus matched controls without a VPD diagnosis.

## Methods

### Study design and setting

This retrospective observational study analysed claims data in the hospital setting from adults admitted due to a VPD versus matched controls, using the Optum DOD de-identified Clinformatics Data Mart Database (CDM). The CDM is derived from a database of administrative health claims for members of large commercial and Medicare Advantage health plans (Optum; Optum, Inc, Eden Prairie, Minnesota). Claims are verified, adjudicated and de-identified prior to inclusion. These data are derived from all medical and pharmacy healthcare services claims, with information on healthcare costs and resource utilization. The population is geographically diverse, spanning all 50 states.

**Fig. 1:**
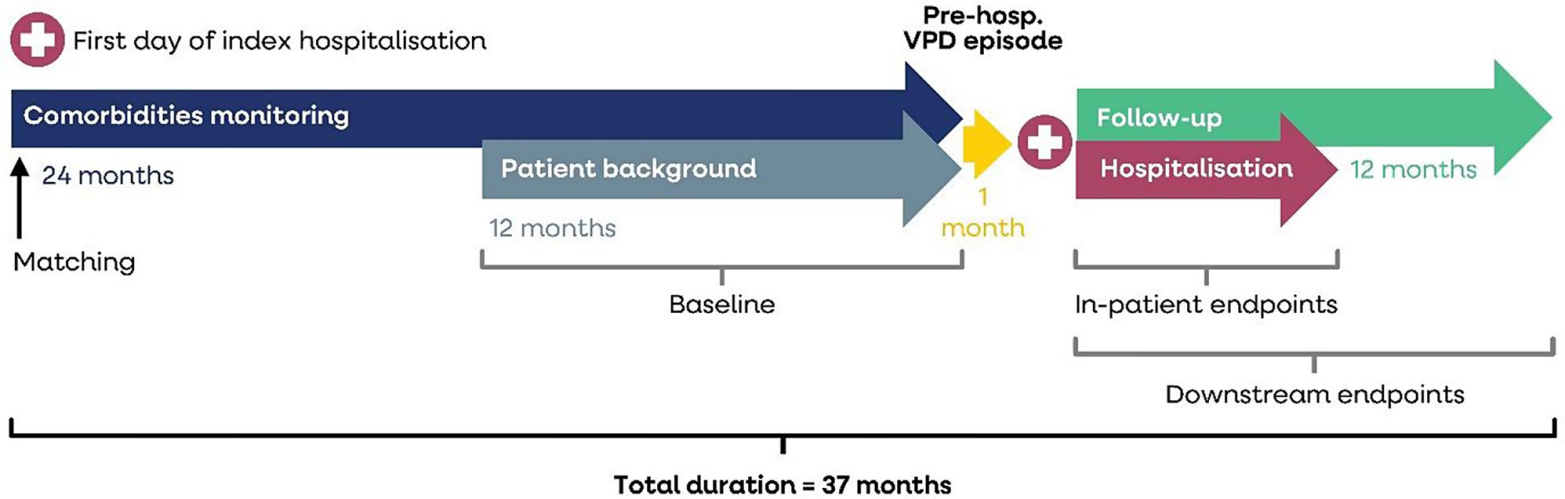
Timeline of index VPD hospitalisation with baseline and follow-up periods

The study period for index hospitalisations was from July 1st, 2016, to June 30th, 2019, to depict epidemiological seasons 2016–2018. The time period was chosen to avoid the potentially disruptive effects on hospital practices caused by the start of the SARS-CoV-2 pandemic. A one year follow-up period after the index VPD hospitalisation was used. The index hospitalisation was the first hospitalisation during the study period where a VPD was diagnosed, as either the principal, secondary or both principal and secondary diagnosis. The baseline period was 25 months, including a 24-month pre-index period to determine underlying comorbidities, and a 1-month monitoring period without hospitalisation prior to the index VPD date (Fig. 1). Thus, continuous enrolment was required, and data were collected from 2014 (i.e., 24 months pre-index hospitalisation) to 2020 (i.e., for 1-year follow-up post discharge). The monitoring period allowed the detection of the onset of the VPD prior to the index date e.g., from potential healthcare resource use in the outpatient setting.

Figure 1 shows the study periods, including 24-month comorbidity monitoring period, 1-month pre-hospitalisation period, index hospitalisation, and 1-year follow-up period.

Current VPDs were defined from the World Health Organization (WHO) list of VPDs [11], and near-term VPDs were defined diseases for which vaccine candidates were in phase III trials on June 1st, 2022. These included vaccines for respiratory syncytial virus (RSV), human immunodeficiency virus (HIV), extraintestinal pathogenic *Escherichia coli* (ExPEC), Chikungunya virus and *Clostridioides difficile* infection.

CDM includes approximately 15–19 million annual lives from all 50 US states, with both commercial and Medicare Advantage claims data, relating to medical and pharmacy claims and inpatient hospital stays.

### Study population

Cases were adults aged 50 years and older, hospitalised on the index date with a primary or secondary VPD diagnosis, or both. Controls had no VPD hospitalisation during the week of, and month prior to, the index date, but could have a VPD hospitalisation outside of this period, or a non-VPD hospitalisation at any time (similar to corresponding cases). The follow-up period for both cases and controls started on the day of the index hospitalisation of the VPD case (which was considered the index date for corresponding controls). Controls who met the inclusion criteria on the day of the index hospitalisation of the VPD case were randomly selected from the CDM population and were matched 4:1 with each case, on covariates including age, sex, ethnicity, insurance status, geographic location, CCI score and healthcare resource use. Baseline values for each covariate were calculated for every quarter over a 24-month period (12-month period for healthcare resource use) prior to the index hospitalisation. Direct matching was applied, on a quarterly basis (see Supplementary file 1 for details on matching). No data on vaccination status were available for cases or controls. The 24-month pre-index monitoring period would also not be sufficient to capture the vaccination status of many vaccines (such as against DTP, HPV, or Zoster) which remain efficacious more than two years after administration. Thus, subjects could be protected by vaccination, while their vaccination status may not be reported in the database.

### Outcome measures

The primary objective was to estimate mortality in cases with a VPD hospitalisation and controls. While secondary objectives included comparing increase in CCI score; onset of new diagnosis of comorbidities; and loss of independence (change in residence status and receipt of home health/care), in cases versus matched controls.

#### Outcomes from downstream effects

The mortality risk ratio (RR) in the VPD group versus controls is presented overall, by age and CCI group, after 30 days and one year. CCI score was used as an overall indicator of health condition. Age and health condition are important factors indicative of frailty, which may confound the effect on mortality and health, but also influence how the patient is taken care of.

The Charlson Comorbidity Index (CCI) score categorises the health status of a patient based on presence and associated weights of diagnosed comorbidities [12]. Cases and controls were matched on baseline CCI scores. The CCI score was assessed throughout the one year follow-up, and the difference in CCI score versus baseline was computed for each patient.

The onset and number of newly diagnosed comorbidities associated with downstream effects of VPDs [1, 5] was determined at baseline and during follow-up. The International Classification of Diseases (ICD)-9 and ICD-10 diagnostic codes of claims were used to estimate the CCI score, whose subscales where used to identify the presence of the following comorbidities: myocardial infarction (MI); congestive heart failure (CHF); cerebrovascular disease (CVD); dementia; chronic pulmonary disease; liver disease (mild, moderate and severe); diabetes with and without chronic complications; renal disease (mild, moderate and severe).

Loss of independence during the follow-up period was a composite endpoint capturing both change in residence status to a long-term care facility, and change to receiving home health/home care, from baseline status. A patient was considered to reside either in a long-term care facility or receive home health/home care, if at least one corresponding claim was recorded during follow-up after the index VPD episode (see Supplementary file 2 for details).

### Statistical analysis

#### Descriptive analysis

A description of the potential disease burden associated with VPD downstream effects within the hospital was computed over a one year follow-up. Outcomes were either directly measured in the claims database (e.g., mortality) or estimated through available proxy-variables (e.g., loss of independence). Means and standard deviations were reported for continuous variables and counts and proportions for dichotomous and categorical variables. Logistic regression was performed for dichotomous variables (mortality, loss of independence, 30-day readmission, onset of new comorbidity); linear regression was performed for continuous variables (direct medical costs, length of stay, time to 1st hospitalisation after discharge, resource utilisation, number of new comorbidities, CCI point increase).

#### Direct comparisons

The potential effect of VPDs, adjusted for covariates such as age, sex and underlying comorbidities, was estimated by means of direct comparisons with matched controls. For continuous variables, the difference in mean values was reported and compared using t-tests (including SE, p value), while for medians, differences were compared using a Wilcoxon test (SE, p value). For the comparison of dichotomous variables, the proportion of the outcome between any two cohorts was stated and the equality of the proportions was tested with a z-test (including SE, p value). The contingency of the outcome and the cohort affiliation was tested with a chi-squared test (including p value). For the comparison of categorical variables, the chi-squared test was used to test the contingency between outcome and cohort affiliation (including p value).

#### Difference-in-differences

The impact of VPDs on health status was assessed by estimating a difference-in-differences (DD) parameter, which generated a before-and-after (difference) comparison for each exposed case and their matched control. Then the difference between both cases and controls was computed (i.e., difference-in-difference) hence providing insights into the difference in magnitude (See Supplementary file 3 for more details). This allowed insights to be developed around the possible extent to which VPDs in older adults may result in a permanent worsening of their health status and ability to recover to baseline function, following a VPD episode as compared with their matched control.

The DD estimator was used to depict different rates of change in outcomes over time between cases and controls. It takes into account baseline differences between cohorts that might have existed before the hospitalisation, and compares the magnitude of change between two points in time (i.e., from the baseline period preceding the index hospitalisation, to the follow-up period after the index hospitalisation [13]).

## Results

Over the study period (mid-2016 to mid-2019), there were 97,057 VPD cases (56.6% female, mean age 76.7 years, 71.1% White, 90.3% with Medicare) and 386,978 matched controls (56.7% female, mean age 76.5 years, 71.2% White, 90.0% with Medicare) (see Table S1 for baseline characteristics).

### Mortality

Mortality rates in the control group were higher than expected at baseline (e.g., from life tables) compared with the general population. The fact of being hospitalized (due to a VPD or other cause) may be indicative of an average worse health status than the general population, which may also translate to the matched controls via the matching procedure. Despite this, overall mortality rates were significantly higher for VPD hospitalised cases versus controls at 30 days follow-up (risk ratio [RR] of 4.08 [95% CI 3.98–4.18]) and at one year follow-up (RR 2.76 [2.73–2.80]). The largest effects (RR) of a VPD episode on mortality were in younger and healthier subgroups (Table 1).

**Table 1:** Mortality risk ratio for VPD cases versus controls at 30 days and one year, stratified by age and CCI group

	Mortality risk ratio (RR)	
	RR (95% CI) at 30 days	RR (95% CI) at one year
Overall	**4.08** (3.98–4.18)	**2.76** (2.73–2.80)
Age group: 50–64	**6.32** (5.69–7.01)	**4.00** (3.79–4.23)
Age group: 65–79	**5.09** (4.87–5.31)	**3.55** (3.47–3.64)
Age group: 80+	**3.51** (3.41–3.62)	**2.33** (2.29–2.37)
CCI group: 0	**7.60** (6.67–8.65)	**6.26** (5.77–6.80)
CCI group: 1–2	**9.91** (9.18–10.70)	**4.69** (4.51–4.88)
CCI group: 3–4	**6.12** (5.86–6.60)	**3.27** (3.17–3.37)
CCI group: 5+	**2.79** (2.71–2.87)	**2.12** (2.09–2.16)

**95% CI**: 95% confidence interval; **CCI**: Charlson Comorbidity Index; **RR**: risk ratio

**Fig. 2:**
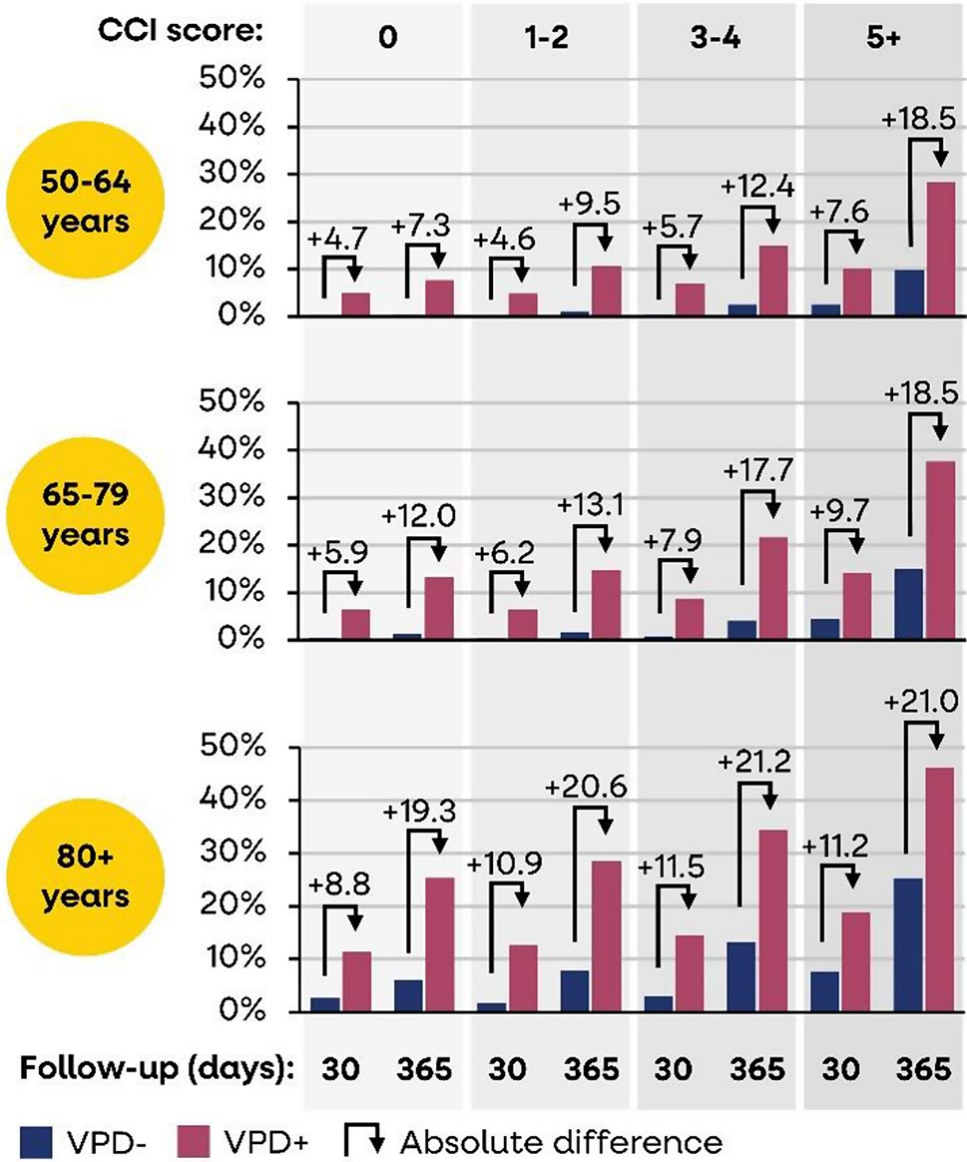
Mortality rate at 30 days and one year follow-up, for VPD cases versus controls, stratified by age and CCI score

Absolute mortality rates tended to increase with age and CCI score as well as over time (more pronounced in the exposed cohort). Mortality rates were significantly higher for VPD patients versus controls in all age groups, CCI score categories, and at 30 days and one year follow-up (Fig. 2). For cases aged 50–54 years without comorbidities (CCI score of 0), mortality rates increased by 4.7 and 7.3% points at 30 days and one year follow-up, respectively, versus controls. And for older cases aged 80 + years with worse health states due to comorbidities (CCI score of 5+), mortality rates increased by 11.2 and 21.0% points at 30 days and one year follow-up, respectively, versus controls.

Figure 2 shows the mortality rate in VPD hospitalised (VPD+) and matched controls (VPD-) by age and CCI score subgroups, at 30 days and 1-year after the VPD hospitalisation.

### Outcomes from VPD downstream effects

#### New comorbidities and increase in CCI score

**Fig. 3:**
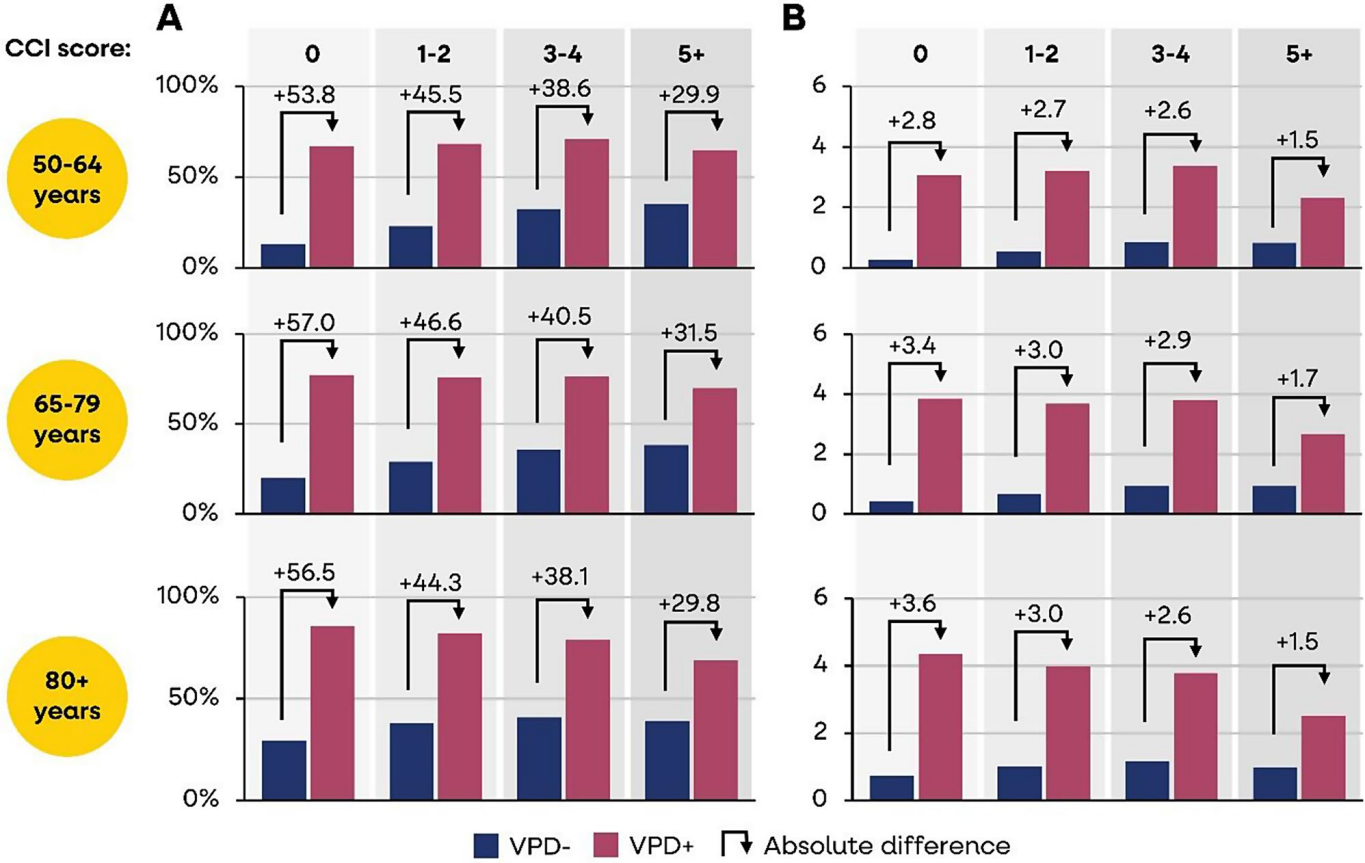
(**a**) Proportion diagnosed with ≥ 1 new comorbidity at one year; (**b**) mean increase in CCI score

Analysis of newly diagnosed comorbidities (beyond those identified within the initial CCI score) showed that significantly more VPD hospitalised cases experienced at least one new comorbidity at one year follow-up compared with matched controls i.e., 65-71.1% versus 13.2–35.1% aged 50–64 years; 69.8–77.1% versus 20.1–38.3% aged 65–79 years; and 68.7–85.8% versus 29.3–40.8% aged 80 + years, all *p* < 0.001 (Fig. 3a). A greater proportion of the older age groups had new diagnoses of comorbidities, while a slightly lower proportion among the CCI 5 + subgroup had new comorbidities, possibly due to multiple comorbidities already present at baseline.

As a result of the onset of new comorbidities, there was an increase in the CCI score at one year follow-up versus baseline, for VPD cases (mean 3.23) versus controls (mean 0.89) i.e., 2.31–3.07 vs. 0.26–0.84 aged 50–64 years; 2.67–3.86 vs. 0.42–0.94 aged 65–79 years; and 2.52–4.35 vs. 0.74–1.15, all *p* < 0.001 (Fig. 3b). The increase in CCI score was slightly lower in individuals with multiple baseline comorbidities (i.e., CCI 5+). Both cases with and without diagnosed comorbidities at baseline experienced an increase in CCI score following the index VPD hospitalisation, with higher average increases in older age groups.

The results show that following the index VPD hospitalisation, cases are more likely to be diagnosed with new comorbidities than their matched controls. Thus, a severe VPD leading to hospitalisation could negatively impact future health status and the chances of recovering to original health states, due to the onset and diagnosis of new comorbidities.

Figure 3a shows the percent with newly diagnosed comorbidities and Fig. 3b shows the mean increase in CCI score, in VPD hospitalised (VPD+) and matched controls (VPD-) by age and CCI score subgroups, at 1-year after the VPD hospitalisation.

#### Loss of independence and change in residence status

**Fig. 4:**
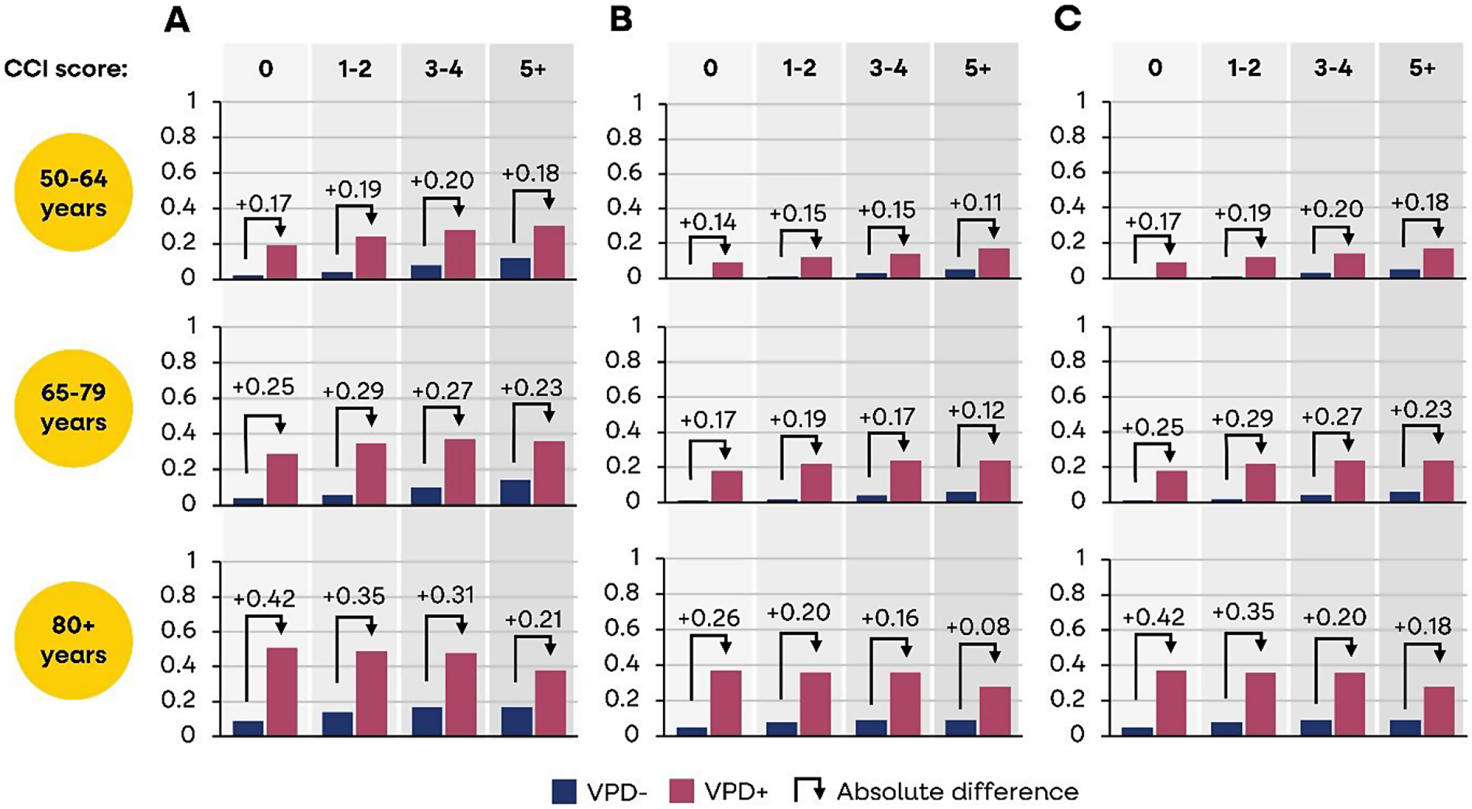
(**a**) New onset of loss of independence, based on (**b**) need for home health/home care; and (**c**) move to long-term care facility

At baseline, around half of cases and controls aged 80 + years with CCI 5 + lived at home without assisted care (Supplementary file 5). At one year after the VPD hospitalisation, significantly more VPD cases (41%) versus controls (12%) experienced some new loss of independence i.e., 25–33% versus 2–12% (range for CCI categories) aged 50–64 years; 36–41% versus 4–13% aged 65–79 years; and 38–56% versus 9–17% aged 80 + years, all *p* < 0.001 (Fig. 4a). The loss of independence was greater among VPD cases than controls (e.g., a greater proportion of cases had a need for home care [Fig. 4b] and a change in residence status to a long-term care facility [Fig. 4c]). Among VPD cases aged 80 + years with CCI 0 (versus controls), 34% (vs. 7%) needed home care and 44% (vs. 5%) had a worsening of residence status (e.g., from living at home to in a long-term care facility), following the index VPD episode (all *p* < 0.001).

Figure 4a shows the composite endpoint **(**percent with loss of independence), based on Fig. 4b (percent needing home health/home care) and Fig. 4c (percent moving into long-term care facility), in VPD hospitalised (VPD+) and matched controls (VPD-) by age and CCI score subgroups, at 1-year after the VPD hospitalisation.

## Discussion

**Fig. 5:**
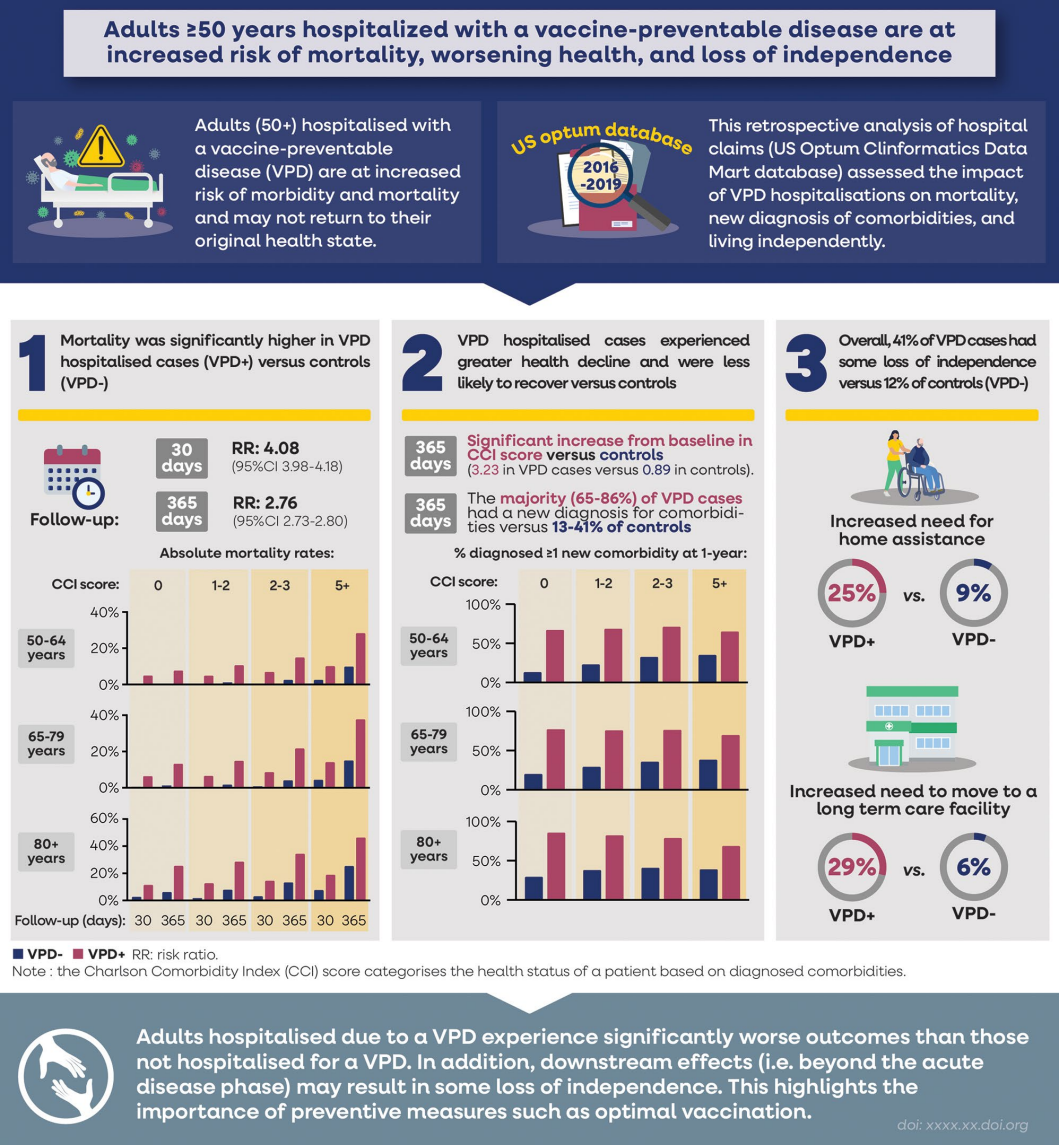
Visual summary

This analysis compared adults aged 50 years and older hospitalised with a VPD diagnosis with matched controls with no VPD hospitalisation, to assess the downstream effects of VPD hospitalisation at and beyond the acute episode. Over the one-year follow-up, in VPD hospitalised patients versus controls, there was an increased mortality risk and worsened health status and daily functioning e.g., leading to loss of independence. A summary of this study is available in Fig. 5.

Mortality rates at 30 days and one year following a VPD hospitalisation were significantly higher versus controls. This premature mortality in adults hospitalised with a VPD represents a large unmet need beyond the acute disease phase, with potential economic implications for society e.g., from loss of activity/productivity. Over the follow-up period, patients hospitalised with a VPD had a significant impact on morbidity: a significantly greater proportion of VPD cases developed new comorbidities; and across subgroups, the mean increase in CCI score points versus baseline was also significantly higher in VPD cases. Therefore, following a VPD hospitalisation, adults experienced a worsening of their health status (i.e., increase in CCI score) during follow-up. Results showed a stronger loss of independence or becoming unable to live independently, among patients hospitalised with a VPD versus controls, with an increased need for assistance such as home care or a long-term care facility. This additional burden on daily functioning can have an important impact on VPD cases. Research on national healthcare spending has shown that the costs of home health care have tripled between 1980 and 2020 (i.e., from 0.9 to 3.0% of total healthcare spending) [14].

Increasing awareness among adults, decision makers and healthcare providers about the benefits of adult vaccination in preventing downstream effects such as mortality, morbidity and loss of independence, is needed. While this study provides some early evidence on the important potential downstream effects of VPD hospitalisation on adult and elderly health, more research is needed to measure and disentangle these impacts of VPDs from other potential confounding factors. For instance, VPD hospitalisation may be a marker for deteriorating health due to diminished immune functioning or other conditions not accounted for in this study, and which may also be associated with various downstream effects. Of note, vaccines can protect against disease burden from VPDs, even with deteriorating immune function. A recent systematic review assessed the burden of VPDs in US adults (aged 19 years and older) at-risk as defined by ACIP, due to higher exposure to VPDs or poorer outcomes due to comorbidities [15]. While this study did not report on long-term downstream effects of infection, the additional burden of VPD episodes versus non-VPDs was reported through increased hospitalisations, durations of inpatient stay and medical costs and a worsening of clinical outcomes. The study highlighted important gaps in the literature on burden of VPDs and stressed the importance of adult life-course vaccination, to reduce the clinical and economic burden to patients, healthcare professionals and the health system [15].

This study has limitations due to the use of claims data and due to the study design. Secondary data from insurance claims databases like CDM lack some clinical variables like vaccination status and disease severity, and do not report non-reimbursed medical services. In addition, since it is based on medical insurance claims, it will not capture the impact of infections that were not serious enough to warrant medical attention. CDM ranks diagnoses made during an inpatient confinement by frequency. In this study, since reason for hospitalisation was not directly available and the aim was to estimate the impact of all VPD hospitalisations, all diagnoses during hospitalisation were considered to identify individuals with a VPD hospitalisation. This may combine consequences of other comorbidities with VPD in the effect of the VPD hospitalisation. Furthermore, limitations around diagnosis of new comorbidities hinder the drawing of robust conclusions about the association of severe VPDs leading to hospitalisation and the outcomes in this study. In the analysis, the identification of a new comorbidity refers to its diagnosis. Thus, if a condition was already present but had not yet been diagnosed at baseline, it would have been counted as a new diagnosis in the study from the moment of diagnosis. This might overestimate the impact of VPDs on onset of new comorbidities, if these subjects were more thoroughly followed up than their controls. Future research using prospective or mixed-method studies could be designed to better capture the true onset of new comorbidities. The use of claims data could also lead to potential bias limiting the generalizability of results. Health insurance coverage in the US is linked to employment and higher socio-economic status [16], while insecure employment is linked with lower socio-economic status [17] which is typically associated with worse health outcomes [18, 19]. The continuous enrolment requirement of this study excluded individuals with gaps in insurance coverage, which may have been due to unsteady employment or worsened health status affecting a change of job or insurance. In 2014 14.4% (27.4 million) of adults under the age of 65 were uninsured at some point during the course of the year [20]. The continuously enrolled study population might, therefore, have a higher socio-economic status, more stable employment and better health and health-seeking behaviours than the general population. In this study, many of the subjects may have been retired from work, and those with Medicare Advantage plans may have a higher socio-economic status than those with Medicare plans or under Medicaid.

This analysis was limited by only assessing the burden of severe VPD episodes that resulted in hospitalisation, whereas less severe VPDs can also contribute to disease burden. Mortality rates in the control group were higher than expected from general population life Table [21]. The health of controls was likely to be slightly worse than the general population because of matching procedures using matching on health-related expenditures and comorbidities. The absolute burden of VPDs, measured by the difference between VPD cases and controls is, therefore, in this regard, likely to be underestimated. Despite the limitations around direct associations between the VPD hospitalisation and the diagnosis of a new comorbidity, however, the findings support the overall hypothesis that downstream effects of VPDs could trigger or exacerbate a comorbidity. To the best of our knowledge, attempting to quantify ‘loss of independence’ using claims data is a new concept from within the current analysis. The concept was defined as a composite of two endpoints, ‘worsening of residence status’ and ‘need for home health’. Results showed a general trend towards worsening of independence status, but further research is needed to ascertain if other events in the one year follow-up may also have contributed to the loss of independence.

## Conclusions

This analysis shows that hospitalised VPD cases suffer significantly worse clinical outcomes than their matched controls with no VPD hospitalisation. The findings suggest the burden of VPD hospitalisation is exacerbated by downstream effects that increase mortality. In addition, US adults may be affected by a loss of independence following VPD hospitalisation, due to a worsening of daily functioning ability.

Evidence on potential downstream effects of infection is relatively limited, and further studies are needed to disentangle the effect of VPDs on new comorbidities versus the natural course of the condition. The study highlights the significant disease burden in patients hospitalised with VPDs after the acute hospitalisation, over a one year follow-up. These downstream effects are not well recognised and often neglected in vaccine policy decision-making. Increasing awareness among adults, healthcare providers and decision makers could help to increase adult vaccination coverage and reduce the clinical and economic burden of VPDs.

## Electronic supplementary material

Below is the link to the electronic supplementary material.

Supplementary Material 1: **Table S1**. Baseline characteristics, **Supplementary file 1**: Matching of cases and controls, **Supplementary file 2**: Loss of independence estimates, Supplementary file 3: Difference in differences measure, **Supplementary file 4**: Mortality, CCI score, and loss of independence results, by age and CCI group, **Supplementary file 5**: Baseline comorbidities and loss of independence

## Data Availability

The datasets generated and analysed during the current study are available from the corresponding author on reasonable request and prior approval from Optum.

## Abbreviations

95% CI: 95 percent confidence interval
ACIP: Advisory Committee on Immunization Practices
CCI: Charlson Comorbidity Index
CDM: Optum’s de-identified Clinformatics Data Mart Database
CHF: Congestive heart failure
COPD: Chronic obstructive pulmonary disease
CVD: Cerebrovascular disease
DD: Difference-in-differences
ExPEC: Extraintestinal pathogenic *Escherichia coli*
HIV: Human immunodeficiency virus
HR: Hazard rate
ICD: International Classification of Diseases
IQR: Interquartile range
MI: Myocardial infarction
RR: Risk Ratio
RSV: Respiratory syncytial virus
US: United States
USD: United States dollar
VPD: Vaccine-preventable disease
WHO: World Health Organization
