# Supplementary material for: Burden of vaccine-preventable diseases in adults (50+) in the United States: a retrospective claims analysis
Source: BMC Public Health. 2024 Oct 25;24:2960. doi: 10.1186/s12889-024-20145-0 (PMC11515361; doi:10.1186/s12889-024-20145-0)

## Table S1. Baseline characteristics in cases and controls

|  |  | **Cases**  **n (%)** | **Controls**  **n (%)** | **SMD/ Chi-2 p-value^a^** |
| --- | --- | --- | --- | --- |
| Total |  | 97057 (100) | 386978 (100) |  |
| Age | Mean, SD | 76.7 (9.6) | 76.5 (9.3) | 0.02 |
|  | Median, IQR | 78.0 (14.0) | 78.0 (14.0) |  |
|  | Q1, Q3 | 71.0 (85.0) | 71.0 (85.0) |  |
|  | Min, Max | 50 (90) | 50 (90) |  |
| Age category | 50-64 | 12219 (12.59) | 46747 (12.08) | <0.01 |
|  | 65-80 | 44681 (46.04) | 175621 (45.38) |  |
|  | 81+ | 40157 (41.37) | 164610 (42.54) |  |
| Sex | Female | 54967 (56.63) | 219267 (56.66) | <0.01 |
|  | Male | 42069 (43.34) | 167659 (43.33) |  |
|  | Unknown/missing | 21 (0.02) | 52 (0.01) |  |
| Race | Asian | 2727 (2.81) | 10740 (2.78) | 0.93 |
|  | Black | 11240 (11.58) | 44689 (11.55) |  |
|  | Hispanic | 10025 (10.33) | 39921 (10.32) |  |
|  | Unknown/missing | 4101 (4.23) | 16197 (4.19) |  |
|  | White | 68964 (71.06) | 275431 (71.17) |  |
| Health plan type | Exclusive/Preferred provider organization | 7602 (7.83) | 31576 (8.16) | <0.01 |
|  | Health Maintenance organization | 24462 (25.20) | 99368 (25.68) |  |
|  | Indemnity | 1556 (1.60) | 6181 (1.60) |  |
|  | Other | 57838 (59.59) | 226480 (58.53) |  |
|  | Point of service | 5599 (5.77) | 23373 (6.04) |  |
| Payer type | Commercial | 9439 (9.73) | 38577 (9.97) | <0.01 |
|  | Medicare | 87618 (90.27) | 348401 (90.03) |  |
| Geographic region | Midwest | 22781 (23.47) | 90662 (23.43) | 0.97 |
|  | Northeast | 13723 (14.14) | 54450 (14.07) |  |
|  | South | 35890 (36.98) | 143334 (37.04) |  |
|  | Unknown/missing | 88 (0.09) | 365 (0.09) |  |
|  | West | 24575 (25.32) | 98167 (25.37) |  |
| Cost category |  | 18 (0.02) | 22 (0.01) | <0.01 |
|  | 0-<Q1 | 18089 (18.64) | 89813 (23.21) |  |
|  | Median-<Q3 | 25468 (26.24) | 97524 (25.20) |  |
|  | Q1-<Median | 22884 (23.58) | 99624 (25.74) |  |
|  | Q3+ | 30598 (31.53) | 99995 (25.84) |  |
| CCI category | 0-<Q1 | 7834 (8.07) | 35087 (9.07) | <0.01 |
|  | Median-<Q3 | 34906 (35.96) | 139138 (35.96) |  |
|  | Q1-<Median | 23921 (24.65) | 101124 (26.13) |  |
|  | Q3+ | 30396 (31.32) | 111629 (28.85) |  |
| Myocardial Infarction | 0 | 81870 (84.35) | 333553 (86.19) | 0.05 |
|  | 1 | 15187 (15.65) | 53425 (13.81) |  |
| Congestive Heart Failure | 0 | 62723 (64.62) | 281258 (72.68) | 0.18 |
|  | 1 | 34334 (35.38) | 105720 (27.32) |  |
| Cerebrovascular Disease | 0 | 72966 (75.18) | 291864 (75.42) | <0.01 |
|  | 1 | 24091 (24.82) | 95114 (24.58) |  |
| Dementia | 0 | 77547 (79.90) | 325004 (83.99) | 0.11 |
|  | 1 | 19510 (20.10) | 61974 (16.01) |  |
| Chronic pulmonary disease | 0 | 52447 (54.04) | 237094 (61.27) | 0.15 |
|  | 1 | 44610 (45.96) | 149884 (38.73) |  |
| Mild Liver Disease | 0 | 87644 (90.30) | 353013 (91.22) | 0.03 |
|  | 1 | 9413 (9.70) | 33965 (8.78) |  |
| Diabetes without complications | 0 | 55468 (57.15) | 227480 (58.78) | 0.03 |
|  | 1 | 41589 (42.85) | 159498 (41.22) |  |
| Renal (Mild or Moderate) | 0 | 67493 (69.54) | 276822 (71.53) | 0.04 |
|  | 1 | 29564 (30.46) | 110156 (28.47) |  |
| Diabetes with complications | 0 | 97057 (100) | 386974 (100) | <0.01 |
|  | 2 |  | 4 (0.00) |  |
| Moderate or Severe Liver Disease | 0 | 95025 (97.91) | 381833 (98.67) | 0.06 |
|  | 3 | 2032 (2.09) | 5145 (1.33) |  |
| Renal severe | 0 | 87697 (90.36) | 361798 (93.49) | 0.12 |
|  | 3 | 9360 (9.64) | 25180 (6.51) |  |

^a^ SMD = Standardized Mean Difference, computed for continuous or bimodal variables and a value >0.20 means there is a difference statistically significant between both cohorts and a Chi-2 p-value is computed for categorical variables with at least 3 modalities and a value < 0.05 means there is a difference statistically significant between both cohorts.

**Supplementary file** 1**: Matching of cases and controls**

To analyse the incremental burden and health outcomes associated with VPDs, VPD-hospitalised cases were compared to non-VPD hospitalised controls. To enable a meaningful comparison between these groups and to diminish the influence of underlying difference between cohorts, direct matching was applied. With this approach four control subjects were matched with each VPD index case (1:4). The following demographic and clinical covariates were included:

- Age category (at the end of the quarterly reference baseline period)
- Sex (at the end of the quarterly reference baseline period)
- Geographic region (at the end of the quarterly reference baseline period as available)
- Race/ethnicity (at the end of the quarterly reference baseline period as available)
- Health plan type (at the end of the quarterly reference baseline period as available)
- Payer type (at the end of the quarterly reference baseline period as available)
- CCI score (observed during the 24-month quarterly reference baseline period)
- All-cause healthcare costs (during the 12-month quarterly reference baseline period, categorized in 3 categories: low, medium, and high).

**Figure S1. Graphical representation of matching mechanism**


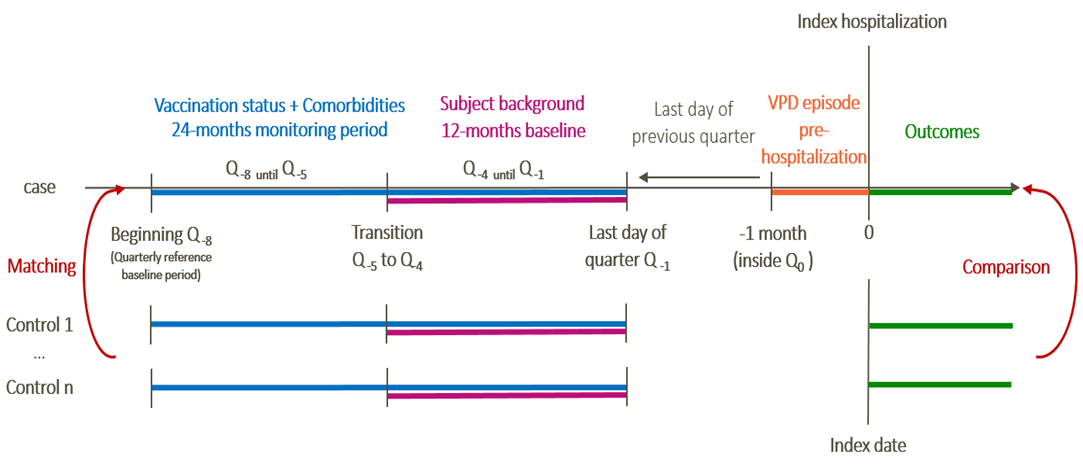


Cases with a VPD hospitalisation and their controls were matched on a quarterly basis, which created a time lag of up to 3 months for the calculation of the baseline values on which the cohorts were matched and the actual index date. The time difference between the matching and the evaluation of endpoints could potentially lead to bias, especially if the two cohorts have different rates of change regarding outcomes. To detect potential differences between the matched cohorts (matched at the end of the quarter prior to the VPD episode) that might have emerged at the beginning of the VPD episode, balance checks were performed. Potential imbalances between the matched cohorts (matched at the end of the quarter prior to the VPD episode) that might have emerged at the beginning of the VPD episode, were evaluated using standardized mean differences and Chi-square tests. No marked imbalance were found to justify the inclusion of these variables in the regression analysis model.

## Supplementary file 2: Loss of independence estimates

Loss of independence was a composite endpoint capturing both change in residence status ‘long-term care facility’ as well as change in home health/home care status. A patient was considered to reside either in a long-term care facility or receiving home health/home care if there was at least one corresponding claim recorded.

- Residence status was approximated by type of inpatient or outpatient claim which was determined following the algorithm of the Stanford Center of Population Health Sciences (Stanford Medicine, 2022) considering the variables Place of Service (POS) (Assisted living facility, skilled nursing facility, nursing facility or custodial care facility) and the variable type of service (TOS_CD) (Hospice Visits, Rehab/Skilled nursing facilities).
- Home health/home care status was approximated by CPT and HCPCS codes for claims of the patient relating to home health/home care services.

## Supplementary file 3: Difference in differences measure

Difference-in-differences requires data on outcomes in the cohort of interest (that had a VPD hospitalisation, A) and the control cohort (C) – both at baseline and follow-up period. The difference-in-differences estimator assumes a parallel trend (in the absence of an index hospitalisation, both groups would have trended equally). Due to the matching procedure on relevant clinical and demographic covariates, this assumption is likely to hold. Simple computation of the difference-in-differences without covariates could be conducted as follows:

1. Calculation of the before-after difference in the outcome (Y) for the VPD cohort (A_episode – A_baseline).
2. Calculation of the before-after difference in the outcome (Y) for the control cohort (C_episode – C_baseline)
3. Calculating the difference between the difference in outcomes for the VPD cohort (A) and the comparison cohort (C). This is the difference-in-differences: (DD)=(A_episode-A_baseline)-(C_episode-C_baseline).

The estimation equation for the difference-in-differences estimator is given by:

$\gamma_{it}= \beta_{0}+ \beta_{1}P_{t}+ \beta_{2}T_{i} + \beta_{3}\left( P_{t}* T_{i} \right)+ \delta X_{i}+ \varepsilon_{it}$

where $\gamma_{it}$ is the outcome of individual i at time t, $P_{t}$ is a dummy variable for the time period (0 = baseline, 1 = episode), $T_{i}$ is a dummy variable for the treatment cohort (0 = control cohort, 1= treatment cohort), $X_{i}$ is a vector of included control variables and $\varepsilon_{it}$ states the random error term. The difference-in-differences estimator is given by parameter $\beta_{3}$. This is the difference in the change rate (between T0 and T1) between both cohorts (between-cohort difference). The within-cohort difference is stated by $\beta_{2}$for the control cohort (C) and ($\beta_{2}$+ $\beta_{3}$) for the VPD cohort (A)

Demographic and clinical covariates were included in the model, even though the cohorts were already matched by these, to confirm robustness.

## Supplementary file 4: Mortality, CCI score, and loss of independence results, by age and CCI group

**Table S2. Mortality analysis for 30 and 365 days - stratified by age and CCI group**

| Strata | 30 days |  | 365 days |  |
| --- | --- | --- | --- | --- |
| Overall | P(exposed):  P(unexposed):  RR:  OR:  OR*: | 0.124  0.030  **4.083** (3.984; 4.184)  4.520 (4.402; 4.641)  4.076 (3.957; 4.199) | P(exposed):  P(unexposed):  RR:  OR:  OR*: | 0.306  0.111  **2.761** (2.725; 2.797)  3.536 (3.477; 3.597)  3.203 (3.141; 3.267) |
| Age group: 50-64 | P(exposed):  P(unexposed):  RR:  OR:  OR*: | 0.074  0.012  **6.318** (5.691; 7.014)  6.740 (6.050; 7.514)  5.498 (4.895; 6.174) | P(exposed):  P(unexposed):  RR:  OR:  OR*: | 0.187  0.047  **4.004** (3.789; 4.231)  4.687 (4.412; 5.000)  3.846 (3.588; 4.122) |
| Age group: 65-79 | P(exposed):  P(unexposed):  RR:  OR:  OR*: | 0.104  0.020  **5.087** (4.869; 5.314)  5.561 (5.309; 5.826)  4.564 (4.338; 4.802) | P(exposed):  P(unexposed):  RR:  OR:  OR*: | 0.266  0.075  **3.552** (3.470; 3.635)  4.479 (4.353; 4.608)  3.697 (3.580; 3.818) |
| Age group: 80+ | P(exposed):  P(unexposed):  RR:  OR:  OR*: | 0.158  0.045  **3.514** (3.408; 3.623)  3.984 (3.850; 4.123)  3.515 (3.380; 3.655) | P(exposed):  P(unexposed):  RR:  OR:  OR*: | 0.376  0.161  **2.330** (2.292; 2.368)  3.131 (3.059; 3.204)  2.710 (2.638; 2.785) |
| CCI group: 0 | P(exposed):  P(unexposed):  RR:  OR:  OR*: | 0.078  0.010  **7.597** (6.674; 8.648)  8.157 (7.135; 9.337)  10.912 (9.398; 12.67) | P(exposed):  P(unexposed):  RR:  OR:  OR*: | 0.164  0.026  **6.261** (5.766; 6.799)  7.291 (6.662; 7.979)  9.183 (8.273; 10.19) |
| CCI group: 1-2 | P(exposed):  P(unexposed):  RR:  OR:  OR*: | 0.090  0.009  **9.908** (9.177; 10.698)  10.788 (9.968; 11.68)  11.396 (10.41; 12.48) | P(exposed):  P(unexposed):  RR:  OR:  OR*: | 0.203  0.043  **4.692** (4.514; 4.877)  5.631 (5.388; 5.885)  5.610 (5.325; 5.910) |
| CCI group: 3-4 | P(exposed):  P(unexposed):  RR:  OR:  OR*: | 0.112  0.018  **6.124** (5.855; 6.595)  6.873 (6.451; 7.321)  6.445 (5.989; 6.936) | P(exposed):  P(unexposed):  RR:  OR:  OR*: | 0.272  0.083  **3.269** (3.172; 3.368)  4.116 (3.964; 4.274)  3.758 (3.597; 3.926) |
| CCI group: 5+ | P(exposed):  P(unexposed):  RR:  OR:  OR*: | 0.156  0.056  **2.786** (2.705; 2.870)  3.117 (3.014; 3.224)  2.787 (2.687; 2.891) | P(exposed):  P(unexposed):  RR:  OR:  OR*: | 0.402  0.189  **2.123** (2.091; 2.156)  2.876 (2.811; 2.943)  2.515 (2.452; 2.579) |

**Table S3. Increase in CCI score - stratified by age and CCI group**

| age | CCI | N | Exposed | | Unexposed | | mean (exp)-mean (unexp) | lower CI | upper CI |
| --- | --- | --- | --- | --- | --- | --- | --- | --- | --- |
|  |  |  | Mean | SD | Mean | SD |  |  |  |
| all | all | 484,035 | 3.23 | 3.51 | 0.89 | 1.91 | 2.34 | 2.32 | 2.36 |
| 50-64 | all | 58,966 | 2.83 | 3.39 | 0.67 | 1.64 | 2.16 | 2.10 | 2.23 |
| 50-64 | CCI: 0 | 8,513 | 3.07 | 3.81 | 0.26 | 0.96 | 2.82 | 2.63 | 3.01 |
| 50-64 | CCI: 1-2 | 14,988 | 3.21 | 3.68 | 0.54 | 1.50 | 2.67 | 2.54 | 2.81 |
| 50-64 | CCI: 3-4 | 11,974 | 3.38 | 3.67 | 0.84 | 1.89 | 2.55 | 2.39 | 2.70 |
| 50-64 | CCI: 5+ | 23,491 | 2.31 | 2.85 | 0.83 | 1.75 | 1.49 | 1.40 | 1.57 |
| 65-79 | all | 206,689 | 3.26 | 3.59 | 0.82 | 1.82 | 2.45 | 2.41 | 2.48 |
| 65-79 | CCI: 0 | 19,298 | 3.86 | 4.20 | 0.42 | 1.27 | 3.44 | 3.30 | 3.58 |
| 65-79 | CCI: 1-2 | 54,449 | 3.69 | 3.84 | 0.66 | 1.63 | 3.03 | 2.95 | 3.10 |
| 65-79 | CCI: 3-4 | 48,057 | 3.81 | 3.81 | 0.94 | 2.01 | 2.87 | 2.79 | 2.95 |
| 65-79 | CCI: 5+ | 84,885 | 2.67 | 3.11 | 0.94 | 1.91 | 1.73 | 1.69 | 1.78 |
| 80+ | all | 218,380 | 3.31 | 3.46 | 1.02 | 2.05 | 2.30 | 2.26 | 2.33 |
| 80+ | CCI: 0 | 15,110 | 4.35 | 3.87 | 0.74 | 1.82 | 3.61 | 3.46 | 3.76 |
| 80+ | CCI: 1-2 | 55,608 | 3.99 | 3.72 | 1.00 | 2.04 | 3.00 | 2.92 | 3.07 |
| 80+ | CCI: 3-4 | 59,108 | 3.78 | 3.59 | 1.15 | 2.23 | 2.63 | 2.56 | 2.70 |
| 80+ | CCI: 5+ | 88,554 | 2.52 | 2.97 | 0.98 | 1.95 | 1.54 | 1.49 | 1.58 |

**Table S4. New loss of independence - stratified by age and CCI group**

| age | CCI | N | Exposed | | Unexposed | | mean (exp)-mean (unexp) | lower CI | upper CI |
| --- | --- | --- | --- | --- | --- | --- | --- | --- | --- |
|  |  |  | Mean | SD | Mean | SD |  |  |  |
| all | all | 484,035 | 0.41 | 0.49 | 0.12 | 0.33 | 0.29 | 0.29 | 0.29 |
| 50-64 | all | 58,966 | 0.30 | 0.46 | 0.08 | 0.26 | 0.23 | 0.22 | 0.24 |
| 50-64 | CCI: 0 | 8,513 | 0.25 | 0.43 | 0.02 | 0.14 | 0.23 | 0.21 | 0.25 |
| 50-64 | CCI: 1-2 | 14,988 | 0.29 | 0.45 | 0.04 | 0.20 | 0.25 | 0.23 | 0.26 |
| 50-64 | CCI: 3-4 | 11,974 | 0.33 | 0.47 | 0.08 | 0.27 | 0.25 | 0.23 | 0.27 |
| 50-64 | CCI: 5+ | 23,491 | 0.32 | 0.47 | 0.12 | 0.32 | 0.20 | 0.19 | 0.22 |
| 65-79 | all | 206,689 | 0.39 | 0.49 | 0.10 | 0.30 | 0.29 | 0.29 | 0.29 |
| 65-79 | CCI: 0 | 19,298 | 0.36 | 0.48 | 0.04 | 0.19 | 0.33 | 0.31 | 0.34 |
| 65-79 | CCI: 1-2 | 54,449 | 0.40 | 0.49 | 0.06 | 0.24 | 0.34 | 0.33 | 0.35 |
| 65-79 | CCI: 3-4 | 48,057 | 0.41 | 0.49 | 0.10 | 0.30 | 0.31 | 0.30 | 0.32 |
| 65-79 | CCI: 5+ | 84,885 | 0.37 | 0.48 | 0.13 | 0.34 | 0.24 | 0.23 | 0.25 |
| 80+ | all | 218,380 | 0.46 | 0.50 | 0.15 | 0.36 | 0.31 | 0.30 | 0.31 |
| 80+ | CCI: 0 | 15,110 | 0.56 | 0.50 | 0.09 | 0.29 | 0.47 | 0.45 | 0.49 |
| 80+ | CCI: 1-2 | 55,608 | 0.53 | 0.50 | 0.14 | 0.35 | 0.39 | 0.38 | 0.40 |
| 80+ | CCI: 3-4 | 59,108 | 0.50 | 0.50 | 0.17 | 0.37 | 0.33 | 0.32 | 0.34 |
| 80+ | CCI: 5+ | 88,554 | 0.38 | 0.49 | 0.17 | 0.37 | 0.22 | 0.21 | 0.22 |

## Supplementary file 5: Baseline data for loss of independence


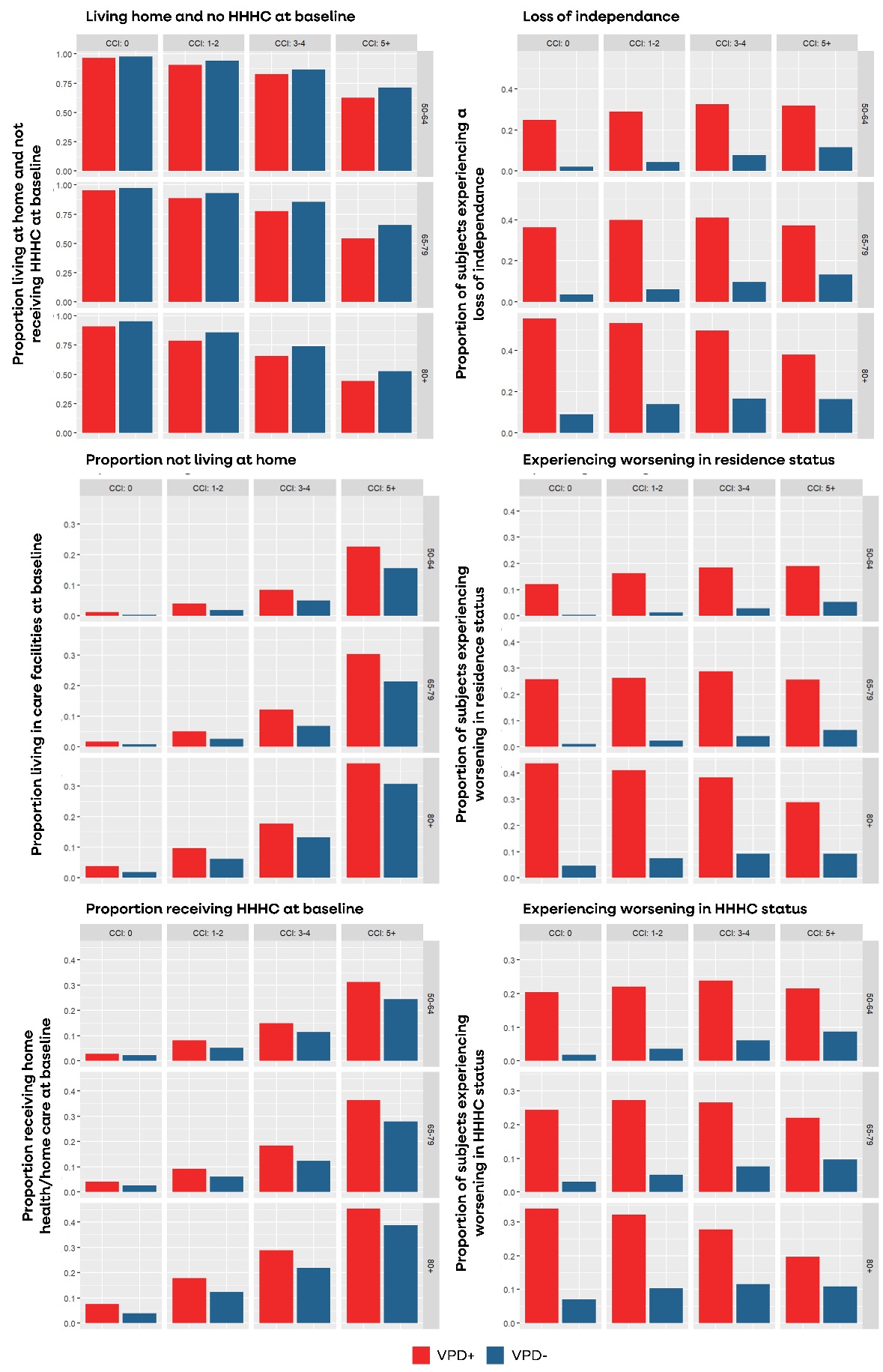

Supplement: Supplementary file 1 — Supplementary Material 1: Table S1. Baseline characteristics, Supplementary file 1: Matching of cases and controls, Supplementary file 2: Loss of independence estimates, Supplementary file 3: Difference in differences measure, Supplementary file 4: Mortality, CCI score, and loss of independence results, by age and CCI group, Supplementary file 5: Baseline comorbidities and loss of independence [file 12889_2024_20145_MOESM1_ESM.docx]
